# Integrating network pharmacology, molecular docking and experimental verification to reveal the mechanism of artesunate in inhibiting choroidal melanoma

**DOI:** 10.3389/fphar.2024.1448381

**Published:** 2024-08-09

**Authors:** Qing-yue Ma, Yi-chong Liu, Qian Zhang, Wen-dan Yi, Ying Sun, Xiao-di Gao, Xin-tong Zhao, Hao-wen Wang, Ke Lei, Wen-juan Luo

**Affiliations:** ^1^ Department of Ophthalmology, The Affiliated Hospital of Qingdao University, Qingdao, China; ^2^ Ophthalmology Department, Qingdao Central Hospital, University of Health and Rehabilitation Sciences (Qingdao Central Hospital), Qingdao, China; ^3^ Tumor Immunology and Cytotherapy of Medical Research Center and Key Laboratory of Pancreatic Disease Clinical Research (Shandong Province), The Affiliated Hospital of Qingdao University, Qingdao, China

**Keywords:** artesunate, choroidal melanoma, anti-cancer mechanism, network pharmacology, molecular docking

## Abstract

**Background:**

Artesunate (ART), a natural compound derived from *Artemisia annua*, has shown promising clinical potentials in the treatment of various tumors, but the exact mechanism is unclear. Choroidal melanoma (CM) is a major malignant ocular tumor in adults, known for its significant malignancy and poor prognosis, with limited efficacy in current treatments. This study explored the anti-CM effects and mechanisms of ART using a combination of network pharmacology, molecular docking and experimental validation.

**Methods:**

Potential targets of ART were screened in PubChem, Swiss Target Prediction and Traditional Chinese Medicine Systems Pharmacology (TCMSP) Database Analysis Platform databases, while target genes related to CM prognosis were selected from Online Mendelian Inheritance in Man (OMIM), GeneCards and DisGeNET databases. The intersection of these two groups of datasets yielded the target genes of ART involved in CM. Protein-protein interaction (PPI) network analysis of the intersecting targets, as well as Gene Ontology (GO) and Kyoto Encyclopedia of Genes and Genomes (KEGG) analyses, were conducted to identify core targets and critical pathways. Molecular docking methods were performed to predict the binding interactions between ART and core targets. The effects of ART on CM were evaluated through CCK8, colony formation, transwell, as well as flow cytometry assays to detect apoptosis, cell cycle, reactive oxygen species (ROS). Western blot (WB) assays were conducted to investigate the impact of ART on key proteins and pathways associated with CM. Finally, *in vivo* assays were conducted to further validate the effects of ART on subcutaneous tumors in nude mice.

**Results:**

Research has shown that key pathways and core targets for ART in treating CM were identified through a network pharmacology approach. Molecular docking results verified the strong binding affinity between ART and these core targets. The analysis and predicted results indicated that ART primarily exerted its effects on CM through various tumor-related pathways like apoptosis. The assays *in vitro* confirmed that ART significantly inhibited the proliferation and migration of CM cells. This was achieved by promoting apoptosis through activation of the p53 signaling pathway, causing cell cycle arrest at the G0/G1 phase by inhibiting the PI3K/AKT/mTOR signaling pathway and increasing the intracellular level of ROS by activating the NRF2/HO-1 signaling pathway. Additionally, the assays *in vivo* further validated the significant proliferation-inhibitory effect of ART on CM.

**Conclusion:**

This study, making the initial exploration, illustrated through network pharmacology combined with molecular docking and *in vitro*/*in vivo* assays, confirmed that ART exerted potential anti-cancer effects on CM by promoting apoptosis, inducing cell cycle arrest and increasing intracellular levels of ROS. These findings suggested that ART held significant therapeutic potential for CM.

## Graphical Abstract

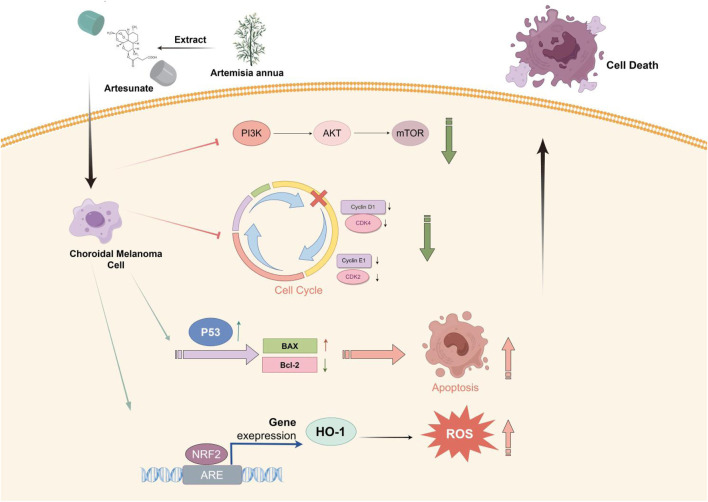

## 1 Introduction

Choroidal melanoma (CM) is a highly malignant tumor with a poor prognosis, patients typically have a median survival time of only about 6 months (Chattopadhyay et al., 2016; Yuan et al., 2022). Currently, the comprehensive clinical treatment strategies for CM mainly include radiation therapy and surgical resection. However, retrospective clinical data analysis indicated that existing clinical treatments had not significantly improved the overall survival rate of CM patients (Chattopadhyay et al., 2016), facing several challenges: severe vision loss or enucleation after surgical resection, damage to surrounding normal tissues due to radiation therapy and poor efficacy of immunotherapy in CM. Therefore, the poor prognosis of CM and the bottlenecks in treatment methods urgently require further research.

Artemisinin has demonstrated significant importance and effectiveness in malaria prevention and control due to its remarkable anti-malarial properties, earning it a Nobel Prize (Luo et al., 2021). In recent years, increasing research has shown that artemisinin also has significant potential in cancer treatment. As a semi-synthetic derivative of artemisinin, ART has been proven to exhibit anti-cancer activity in various cancers. For instance, ART enhanced the sensitivity of esophageal cancer cells to radiation by inhibiting DNA damage repair (Fei et al., 2018), induced autophagy in colon cancer cells (Jiang et al., 2018), promoted apoptosis in chemotherapy-resistant neuroblastoma cells (Michaelis et al., 2010) and induced ferroptosis in head and neck cancer cells to overcome cisplatin resistance (Roh et al., 2016). Therefore, it is speculated that ART may also exerted anti-cancer effects in CM.

As an advantageous tool for identifying medicine targets, network pharmacology has increasingly demonstrated its strengths (Zhao et al., 2023). Its principle involves integrating bioinformatics data and drug databases to help identify potential drug targets, predict drug-target interactions and assess the impact of drugs on disease pathways, thus providing key insights for discovering anti-cancer drugs. Additionally, molecular docking simulation, used in this research, is a widely used molecular modeling technique in drug research that can accurately predict the binding affinity between drugs and potential targets (Pinzi and Rastelli, 2019).

**FIGURE 1:**
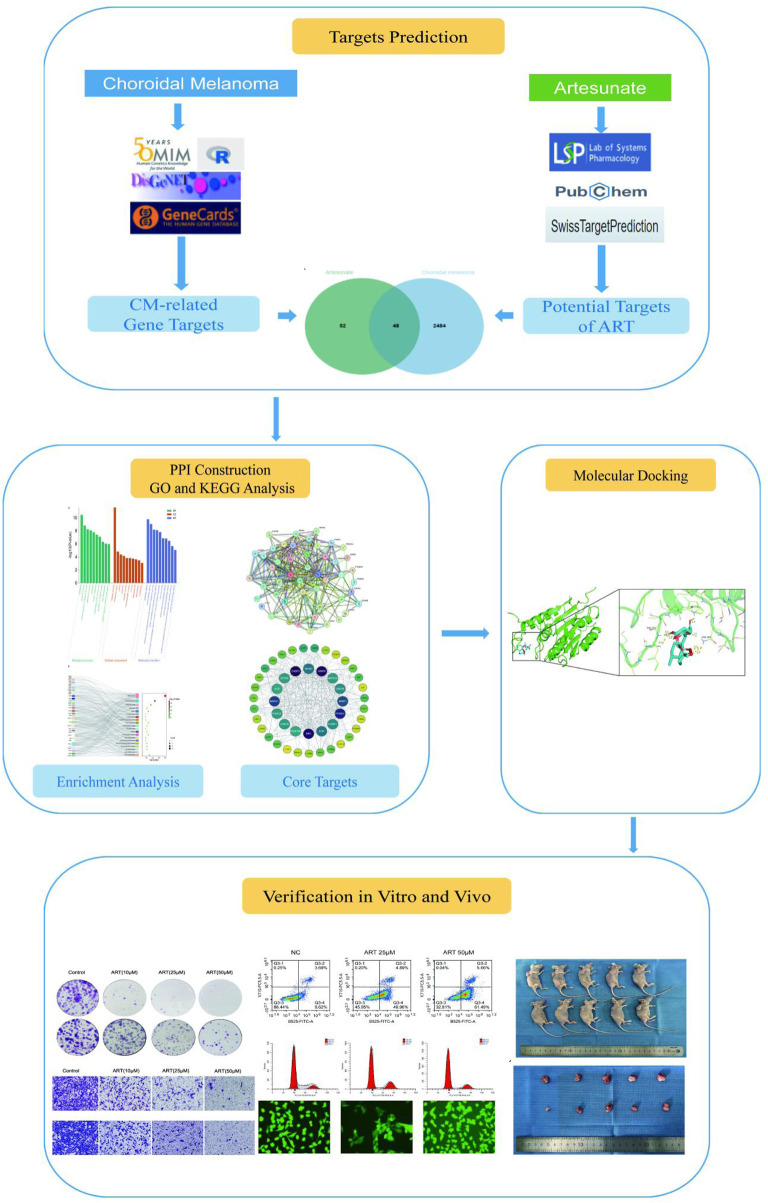
The detailed research process.

This study, with the research process shown in Figure 1, utilized network pharmacology, molecular docking and *in vitro*/*in vivo* assays to demonstrate that ART significantly inhibited the proliferation and migration of CM cells. Furthermore, it validated that the anti-cancer mechanism of ART mainly involved promoting cell apoptosis, inducing cell cycle arrest and enhancing intracellular levels of ROS. This research further established the potential of ART as a novel approach and method for treating CM.

## 2 Materials and methods

### 2.1 Determination of ART treatment targets in CM

Using “artesunate” as a keyword, searched in the PubChem database (https://pubchem.ncbi.nlm.nih.gov/, accessed on 23 September 2023), Swiss Target Prediction database (http://www.swisstargetprediction.ch/, accessed on 23 September 2023) and TCMSP (https://old.tcmsp-e.com/tcmsp.php, accessed on 23 September 2023), obtaining 100 targets related to ART. Then, using “choroidal melanoma” as a keyword, retrieved targets related to CM from the OMIM (https://www.omim.org/, accessed on 23 September 2023), GeneCards database (https://www.genecards.org, accessed on 23 September 2023) and DisGeNET (https://www.disgenet.org, accessed on 23 September 2023), obtaining 2,532 targets associated to CM. Using the NCBI (https://www.ncbi.nlm.nih.gov/, accessed on 23 September 2023) and UniProt databases (https://www.uniprot.org/, accessed on 23 September 2023) to standardize the proteins. Using the Venny 2.1 software (https://bioinfogp.cnb.csic.es/tools/venny, accessed on 23 September 2023) to determine the targets shared between ART and CM, and generated a visualized Venn diagram to illustrate these shared targets.

### 2.2 Construction of PPI network

To explore the interactions among intersecting targets involved in ART’s therapeutic effect against CM and identify key target genes, based on “*homo sapiens*”, using STRING database to construct PPI network and visualized by Cytoscape 3.9.1. The network was established with a PPI score set to 0.9 (highest confidence) to identify closely interacting proteins, and then using CytoNCA plugin to assess the Degree Centrality (DC), Betweenness Centrality (BC) and Closeness Centrality (CC) of network nodes.

### 2.3 GO and KEGG analysis

The target genes were analyzed using the DAVID v6.8 database (http://david.abcc.ncifcrf.gov/, accessed on 23 September 2023) for GO and KEGG analysis (https://www.kegg.jp, accessed on 23 September 2023), comparing them with known biological processes (BP), cellular components (CC) and molecular functions (MF) in GO analysis, and mapping them to pathways for functional annotation and pathway analysis in KEGG analysis. The results were visualized using bar charts and bubble charts provided by the Bioinformatics platform (http://bioinformatics.com.cn/, accessed on 23 September 2023).

### 2.4 Molecular docking

The core targets found in the PPI network were docked with ART structure. The UniProt database contained gene IDs of core targets and Protein Data Bank (PDB, https://www.rcsb.org/) provided 3D structures of proteins. The structure file of ART was imported from the PubChem database into Chem3D software and underwent spatial structure conversion and energy optimization. The AutoDock Vina was used for docking calculations of target proteins and ART, choosing the lowest binding energy in docking results and visualized *via* PyMOL software. Binding energy less than 0 kcal/mol indicates that the ligand can freely bind to the receptor; less than −5 kcal/mol indicates that the ligand has strong affinity with the target protein; less than −7 kcal/mol indicates that the ligand has extremely strong affinity with the target protein (Ge et al., 2023).

### 2.5 Medication

ART and N-acetyl-L-cysteine (NAC) powder were separately purchased from Sigma-Aldrich (A3731; United States of America) and Sigma-Aldrich (PHR1098; United States of America). ML385 powder was sourced from MCE (HY-100523; Shanghai; China). All drugs were dissolved in dimethyl sulfoxide (DMSO) (D8371; Solarbio; Beijing; China) and stored at −80°C. They were diluted to the desired final concentrations in cell culture medium before use. Cell culture medium containing only 0.1% DMSO served as the negative control in the assays.

### 2.6 Cell culture and treatment

The human CM cell lines C918, MUM2B and OMM2.3 were respectively purchased from Procell Life Science and Technology Co., Ltd. (Wuhan; China), Fuheng Biological Technology Co., Ltd. (Shanghai; China) and Saiku Biotechnology Co., Ltd. (Guangzhou; China). The cell line ARPE-19, derived from human retinal pigment epithelium, was provided by the BeNa Culture Collection (Beijing; China). C918, MUM2B and OMM2.3 were grown in RPMI-1640 medium, whereas ARPE-19 was cultured in DMEM medium. All culture media contained 10% fetal bovine serum. The cells were cultured in an incubator (HCP-168; Haier; China) at 37°C with a CO_2_ concentration of 5% and O_2_ concentration of 21%. Cells were passaged when their confluence reached approximately 70%.

### 2.7 CCK-8 assay

Each well of a 96-well plate was seeded with 5,000 cells, after the cells had adhered to the well surface, replaced the cell culture medium with ART in varying concentrations. Each well containing the original medium was incubated for 48 h, and then 10 μL of CCK-8 solution (HY-K0301; MCE; Shanghai; China) was added in it. Following this, the 96-well plate was re-incubated for an additional 1 h. Subsequently, the optical density (OD) of the liquid in each well was measured at a wavelength of 450 nm using an microplate reader (Synergy Neo2; BioTek; United States of America). The OD value reflects the number of viable cells, the more viable cells, the higher the OD value. The OD value at a drug concentration of 0 is set as 100% cell viability, the OD values of each ART-treated group are compared with the control group to calculate the relative viability percentage. Finally, the IC50 value is determined, which is the concentration of the drug that reduces cell viability by 50%.

### 2.8 Colony formation assay

Pretreatment of cells with differing concentrations of ART and/or 5 mM NAC for 24 h was conducted on C918 and MUM2B. Subsequently, 500 cells per well were seeded in a six-well plate and cultured for 14 days. Afterward, fixation and staining were performed using a solution of 4% paraformaldehyde and 0.2% crystal violet. Colonies containing 50 or more cells were counted using ImageJ software for analysis.

### 2.9 Cell migration assay

After a 24 hour-drug pretreatment of C918 and MUM2B cells, cell suspensions were prepared in a serum-free culture medium. Then 4*10^4^ cells were added to the upper chamber (8 μM pore size; Corning; United States of America), a 10% FBS-containing cell culture medium was used as a chemical attractant in the lower chamber, C918 and MUM2B cells were then incubated for an additional 24 h. Cells that migrated to the underside of the membrane were fixed using 4% paraformaldehyde and stained with 0.2% crystal violet. The cells that had crossed the membrane were counted and assessed by ImageJ software.

### 2.10 Apoptosis assay

Early apoptotic cells were labeled by Annexin V-FITC binding to phosphatidylserine on the cell membrane. Propidium iodide (PI) penetrated the cell membrane and can label the nuclei of late apoptotic or dead cells. Using the Annexin V-FITC/PI apoptosis detection kit (E-CK-A211; Elabscience; Wuhan; China) for this part. Cells were pretreated with different concentrations of ART (0, 25, 50 μM) for 24 h and then labeled with the aforementioned reagents. They were analyzed using flow cytometry (CytoFLEX LX; Beckman Coulter; United States of America). The data were processed with FlowJo 7.6 software.

### 2.11 Cell cycle analysis

Cell cycle was analyzed using flow cytometry combined with DNA-binding fluorescent dyes to measure fluorescence intensity. Cells pretreated with different concentrations of ART (0, 25, 50 μM) for 24 h were collected, labeled with PI (E-CK-A351; Elabscience; Wuhan; China) and analyzed using flow cytometry. The data were analyzed using FlowJo 7.6 software.

### 2.12 Measurement of ROS levels

ROS levels were measured using a ROS detection kit (E-BC-K138-F; Elabscience; Wuhan; China) *via* flow cytometry and fluorescence quantification. Cells pretreated with different concentrations of ART (0, 25, 50 μM) for 24 h were washed with serum-free medium, followed by the addition of serum-free medium containing 10 μM DCFH-DA fluorescent probe, incubated for 1 h and then ROS levels were detected using flow cytometry or fluorescence microscopy(Ts2; Nikon; Japan). The data were analyzed using FlowJo 7.6 software.

### 2.13 WB assays

Cells pretreated with different concentrations of ART (0, 25, 50 μM) for 48 h were washed with PBS and lysed with RIPA cell lysis buffer (R0010; Solarbio; Beijing; China) at 4°C for 30 min. Equal concentrations of cell lysates were subjected to SDS-PAGE gel electrophoresis and transferred onto PVDF membranes (IPVH00010; Merck Millipore; United States of America) for WB assays. The primary antibodies were applied and incubated for 14 h at 4°C, the primary antibodies used were as follows: BCL-2(1:1,000; ET1702-53; HUABIO; Hangzhou; China), BAX (1:1,000; ET1603-34; HUABIO; Hangzhou; China), p-PI3K(1:1,000; 4228T; Cell Signaling Technology; MA; United States of America), PI3K(1:1,000; ab191606; Abcam, Cambridge, MA, United Kingdom), p-AKT(1:1,000; ab192623; Abcam, Cambridge, MA, United Kingdom), AKT(1:10,000; ab179463; Abcam, Cambridge, MA, United Kingdom), p-mTOR(1:1,000; T56571S; Abmart; Shanghai; China), mTOR(1:10,000; ab134903; Abmart; Shanghai; China), CyclinD1(1:1,000; ET1601-31; HUABIO; Hangzhou; China), CyclinE1(1:1,000; ER 1906-94; HUABIO; Hangzhou; China), CDK2(1:1,000; ET1602-6; HUABIO; Hangzhou; China), CDK4(1:1,000; ET1612-23; HUABIO; Hangzhou; China), p53(1:1,000; TA0879S; Abmart; Shanghai; China), NRF2(1:1,000; T55136S; Abmart; Shanghai; China), HO-1(1:1,000; 26416S; Cell Signaling Technology; MA; United States of America), GAPDH(1:10,000; 60004-1-Ig; Proteintech; Wuhan; China), α-tubulin(1:10,000; 11224-1-AP; Proteintech; Wuhan; China). Subsequently, the membranes were incubated with secondary antibodies conjugated with horseradish peroxidase (A0208/A0216; Beyotime; China) for 1 h. Target protein bands were detected using ECL reagent (36222ES60; Yeasen; Beijing; China). ImageJ software was used to analyze the grayscale values associated with target protein bands, with the grayscale values of GAPDH/α-Tubulin used as an internal reference.

### 2.14 Subcutaneous xenograft tumor model

The nude mice experiment was approved by the Qingdao University Experimental Animal Welfare Ethics Committee Approval Document. Nude mice were purchased from Beijing Vital River Laboratory Animal Technology Co. Ltd. (Beijing; China) and were acclimated for 1 week. C918 cells (1.5 × 10^6^ cells per mouse) were injected into the right axilla of Balb/c nude mice to establish the subcutaneous xenograft tumor model. Approximately 1 week after injection, palpable tumors were observed in the axillary region of the mice. Randomly divide these mice into two groups, the ART treatment group received daily intraperitoneal injections of ART (75 mg/kg, dissolved in 200 μL of physiological saline), whereas the control group received equal volumes of physiological saline. To observe and measure the subcutaneous tumors, the mice were euthanized following a 14-days treatment period.

### 2.15 Immunohistochemistry (IHC)

Tumor tissues were using 4% paraformaldehyde and paraffin to fix and embed, then sectioned. The sections were subjected to deparaffinization, dehydration and citrate buffer antigen retrieval steps. After these steps, the sections were incubated with the primary antibody Ki67 (1:100; 28074-1-AP; Proteintech; Wuhan; China) at 37°C for 2 h, followed by secondary antibody incubation, DAB staining, hematoxylin counterstaining, dehydration, clearing with xylene and finally mounted.

### 2.16 Statistical analysis and graphical abstract preparation

All experiments must be conducted a minimum of three times unless otherwise specified. The experimental data was subjected to statistical analysis using Prism 8.0 software (GraphPad; United States of America), mainly used unpaired *t*-test and one-way analysis of variance (ANOVA), statistics were calculated by averaging the results of three independent samples and then summarizing them. *p < 0.05, **p < 0.01, ***p < 0.001, ****p < 0.0001. Graphical abstract preparation by Figdraw software.

## 3 Result

### 3.1 Collection of core targets of ART in CM and performing GO and KEGG analysis

**FIGURE 2:**
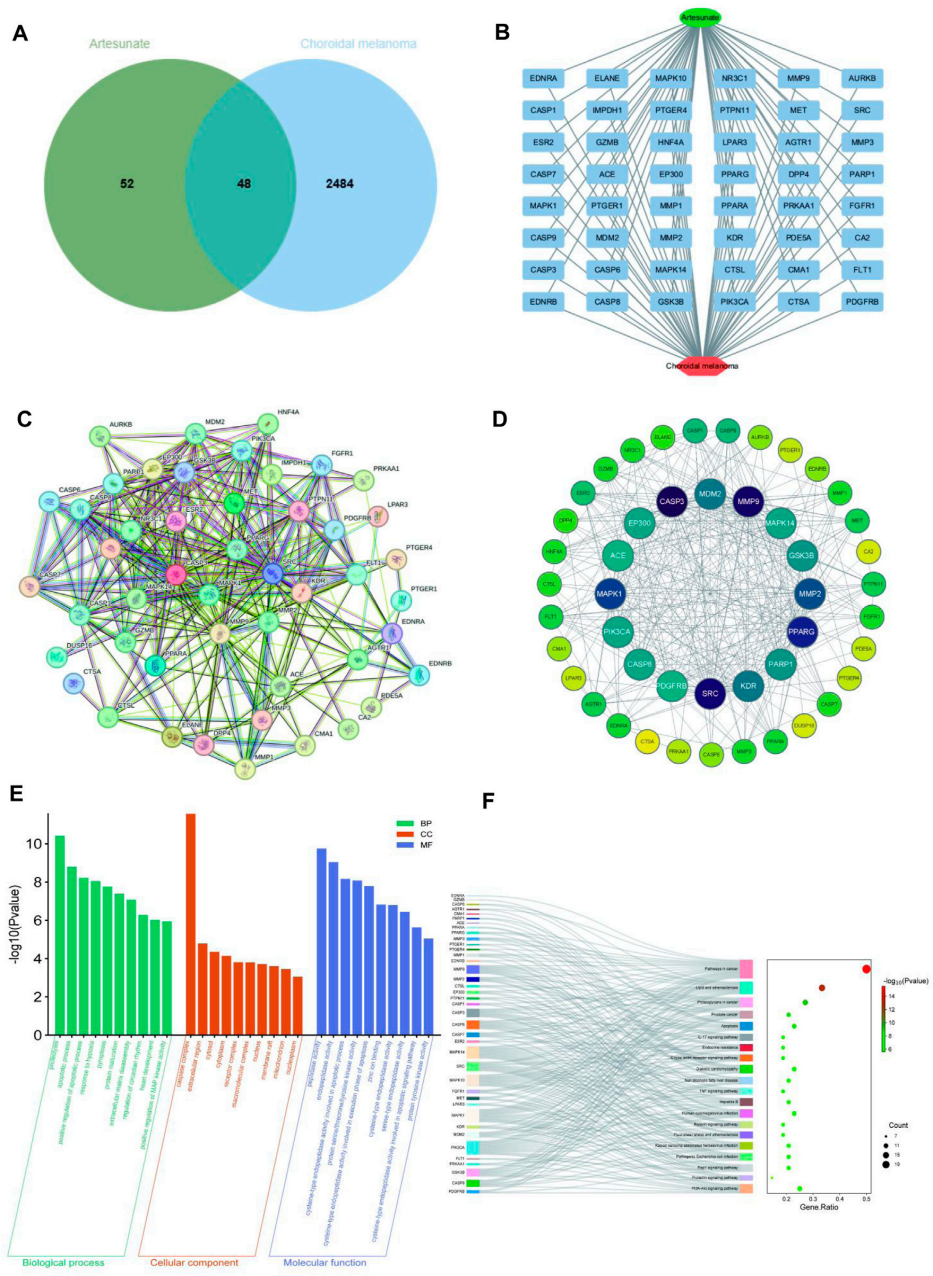
Network pharmacology predicted the core targets and the pathway analysis of the mechanism of ART in CM. **(A)** Venn diagram of common targets of ART in CM. **(B)** Constructing a drug-target-disease network using the 48 common targets of ART in CM. **(C)** Common targets in the PPI network. **(D)** Formulation and presentation of the core targets in the form of a PPI network. **(E)** Top 10 GO terms in BP, CC, and MF categories. **(F)** Top 20 KEGG pathways.

Using information from PubChem, Swiss Target Prediction and TCMSP databases, 100 potential targets of ART were identified. Searching gene targets related to CM from OMIM, GeneCards and DisGeNET databases were combined and deduplicated, and targets with scores ≥ median value were selected as cutoff, resulting in 2,532 disease targets. Intersection analysis was performed between the 100 ART-related target genes and the 2,532 CM-related target genes, yielding 48 intersecting genes considered as potential candidate targets for ART against CM, depicted in a Venn diagram (Figure 2A). Subsequently, a network linking compound, targets and diseases was constructed using Cytoscape 3.9.1, presenting a combined total of 48 gene names involved in drug-disease interactions (Figure 2B). To analyze the interactions between ART and CM intersecting targets, the 48 intersecting targets were analyzed using the STRING database, visualization of the resulting network was carried out using Cytoscape 3.9.1 (Figure 2C). In the PPI network, the lines represented the strength of protein-protein interactions, with darker and more central nodes indicating stronger interactions among targets (Figure 2D). The most central layer represented the core targets in the PPI network, and it was found that these genes are enriched in pathways such as “Pathways in cancer”, “Apoptosis”, “PI3K/AKT signaling pathway” and “p53 signaling pathway” (Supplementary Table S1). This suggested that ART may exerted its anti-CM effects by influencing these tumor-related biological processes in CM.

Enrichment analysis of GO revealed 333 significant GO term entries (*p* < 0.05), encompassing 247 BP, 24 CC and 62 MF (Supplementary Table S1), utilizing bar charts to visualize the top 10 enriched terms in each category (Figure 2E). These results implied that ART might combat CM by modulating the activity of proteins and enzymes within the aforementioned signaling pathways.

Enrichment analysis of KEGG identified significant enrichment in 111 pathways (*p* < 0.05) (Supplementary Table S2). As described in the figure, the top-ranked pathways included several tumor-related pathways such as “Pathways in cancer”, “Apoptosis” and “PI3K/AKT signaling pathway” (Figure 2F). These results suggested that ART might exert its anti-CM effects by influencing crucial pathways and targets related to tumor processes like PI3K/AKT signaling pathway.

Based on the above analyses, it is speculated that ART may exerted its anti-cancer effects by influencing certain tumor cell physiological processes and key molecules. Next, we will further proceed with molecular docking and experimental validation of this prediction.

### 3.2 Molecular docking

In investigating ART’s mechanism in treating CM, we performed molecular docking to simulate the binding configuration between ART and the screened core targets. The results demonstrated that ART exhibited strong binding affinity with molecules in the core targets (all less than −5 kcal/mol), with corresponding binding energy, hydrogen bond sites and bond lengths listed in Table 1.

**TABLE 1:** The binding energy, hydrogen bond sites and bond length of ART with core targets.

Gene	Binding energy (kcal/mol)	Hydrogen bond sites	Hydrogen bond length (Å)
CASP3	−7.1	TRP-214, PHE-250, ASN-208	2.4 Å; 2.1 Å, 2.3 Å, 2.5 Å; 2.1 Å, 2.3 Å, 2.7
PIK3CA	−9.4	ARG-818, HIS-759	2.7 Å, 2.4
MDM2	−6.1	GLN-72	2.9
PPARG	−9.8	ARG-280	2.1
MAPK1	−9.3	ARG-67	2.4
MAPK14	−8.3	LYS-53	3.2
KDR	−7.7	ARG-840	2.6
PDGFRB	−6.6	LYS-366, SER-460	1.9 Å; 2.1 Å, 2.3 Å, 2.1
MMP2	−7	THR-143, LEU-137	2.2 Å; 2.2
MMP9	−6.7	ASN-177, SER-172	2.5 Å; 2.5
PARP1	−7	ARG-330	2.1 Å, 2.1
GSK3B	−8.3	ASP-200, LYS-85	2.2 Å, 2.5
CASP8	−7.5	THR-503	2.1
EP300	−9.8	SER-1400, GLN-1455, LYS-1456	2.6 Å, 2.6 Å; 2.4 Å; 2.3
SRC	−8.1	THR-247, SER-248	1.9 Å, 2.5 Å; 2.0

**FIGURE 3:**
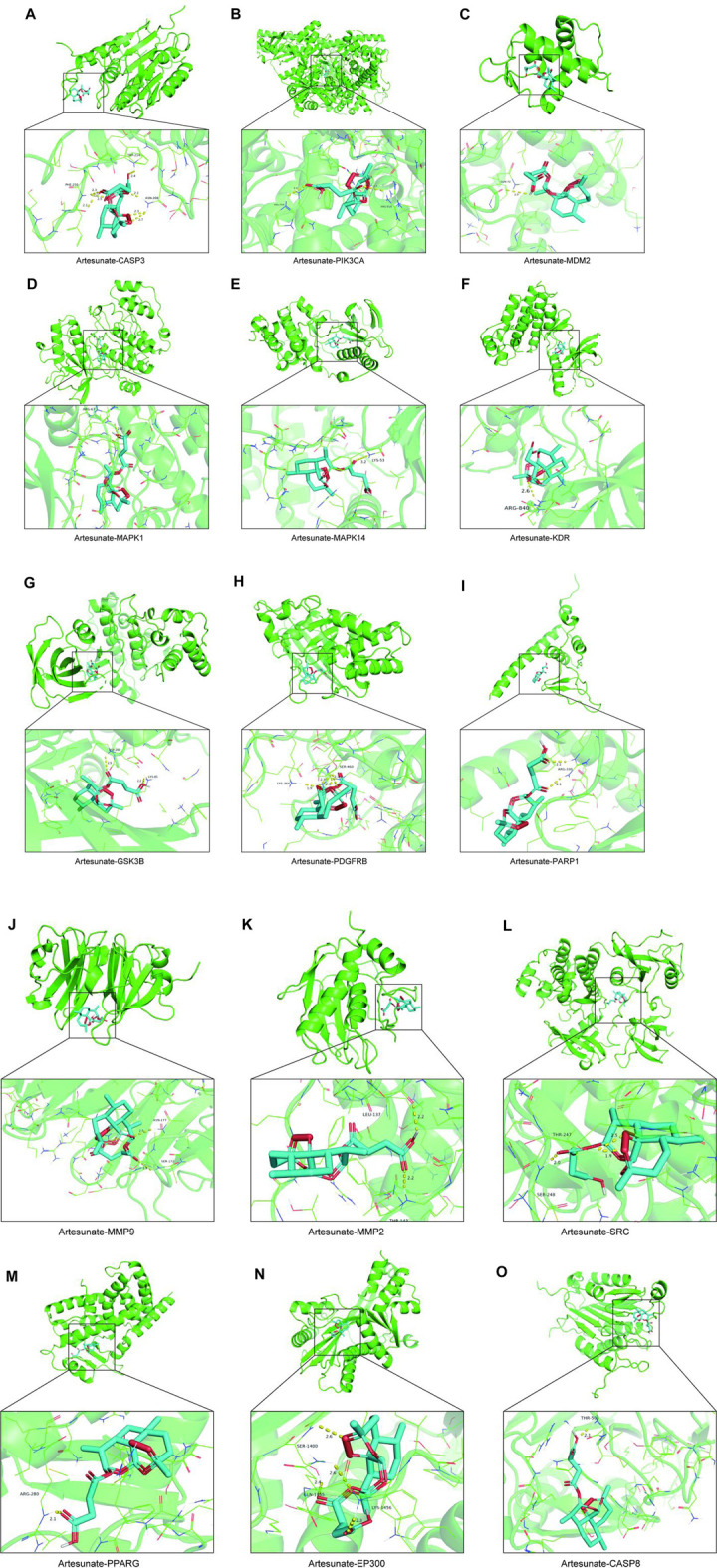
The molecular docking patterns of ART with CASP3, MMP9, PPARG, MAPK1, MMP2, PIK3CA, MDM2, PDGFRB, GSK3B, EP300, SRC, PARP1, MAPK14, KDR, and CASP8.

Moreover, the docking modes of ART with core targets were visualized and presented in Figures 3A–O. It can be observed that ART exhibited strong binding affinity with several genes that play important roles in malignant tumor progression. These genes include like PIK3CA, which is involved in the oncogenic PI3K/AKT/mTOR signaling pathway; MMP9 and MMP2, which promote tumor migration; CASP8 and CASP3, which are involved in the apoptosis process; and MDM2, which regulates the stability of the tumor suppressor gene p53.

Currently, through network pharmacology and molecular docking techniques, we have identified and preliminarily verified the potential pathways and targets by which ART exerted its effects on CM. In the subsequent sections of this article, we conducted a series of experiments to further demonstrate the specific actions and mechanisms by which ART played its role in.

### 3.3 ART inhibited CM cell proliferation and migration in a concentration-dependent manner

**FIGURE 4:**
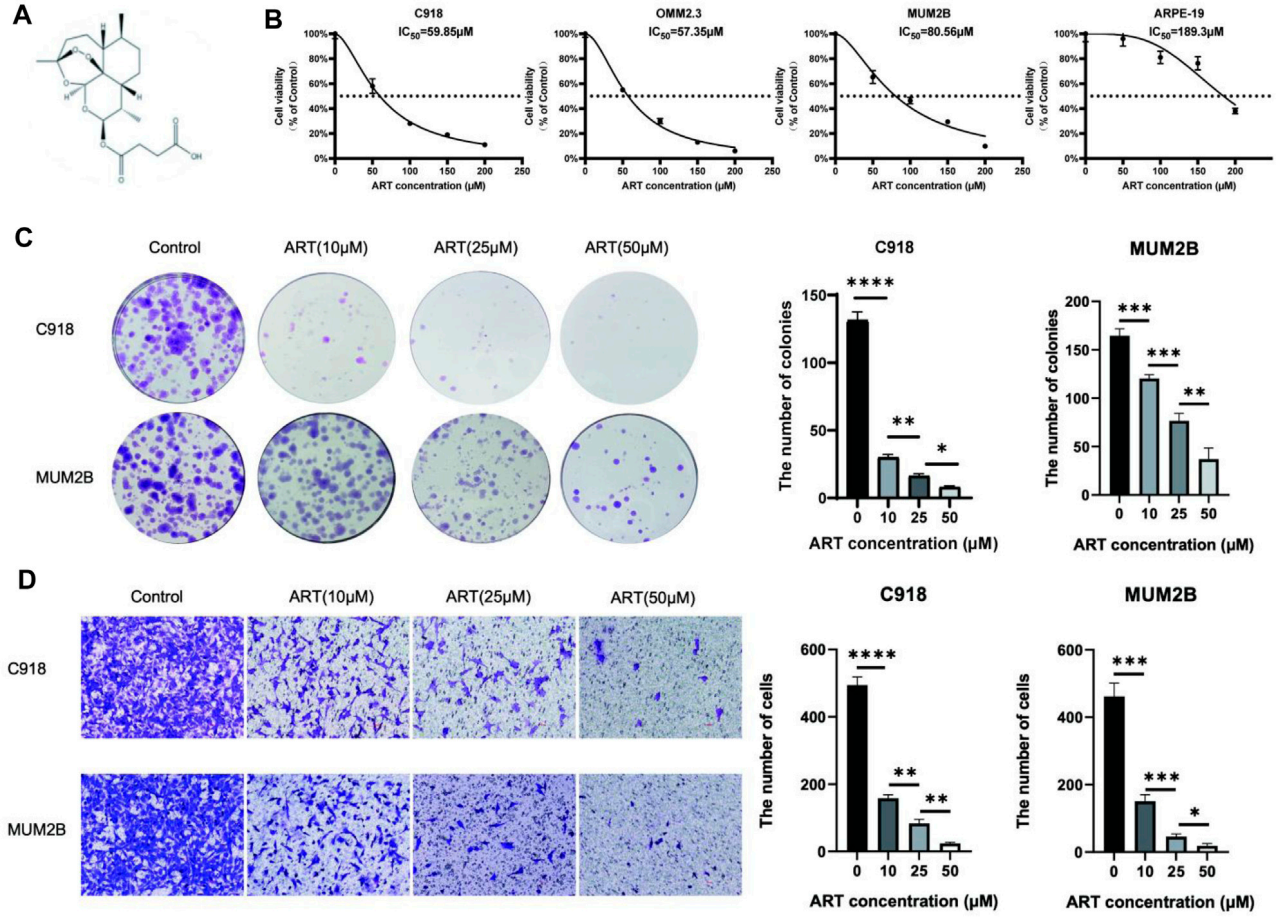
ART inhibited the proliferation and migration of CM cells. **(A)** The chemical structure of ART. **(B)** CCK-8 assay was used to evaluate the viability of CM cells and ARPE-19 following 48 h of exposure to ART at varying concentrations. **(C)** Colony formation assay was used to assess the clonogenic capacity of CM cells after exposure to varying concentrations of ART for 24 h. **(D)** Cell migration assay was used to assess the migration capacity of CM cells after exposure to varying concentrations of ART for 24 h (scale bar = 100 μm).

The chemical structure of ART is shown in Figure 4A. The network pharmacology analysis revealed that ART might have significant anti-cancer effects on CM. Therefore, we conducted preliminary evaluations using CCK-8, colony formation and cell migration assays.

First, to determine the impact of ART on the proliferation of CM cells, we exposed CM cells to various concentrations of ART (0, 50, 150, 200 μM) for 48 h and then measured cell viability using the CCK-8 assay. The results showed that ART inhibited the viability of CM cells in a concentration-dependent manner. Additionally, we investigated the effect of ART on ARPE-19 to differentiate ART’s inhibitory effects from its potential toxicity to retinal normal cells. The IC50, which represents the concentration required to inhibit cell viability by 50%, was found to be 189.3 μM for ART in ARPE-19 (Figure 4B). This was significantly higher than the IC50 of 59.85 μM, 57.35 μM, and 80.56 μM respectively observed in CM cell lines C918, OMM2.3, and MUM2B, indicating that ART has lower toxicity to normal retinal cells.

Next, the colony formation assay revealed that ART inhibited the colony-forming ability of CM cells in a concentration-dependent manner (Figure 4C). These findings suggested that ART exerted a concentration-dependent inhibitory effect on the proliferation of CM cells. Furthermore, inhibiting the migration ability of tumor is crucial for suppressing their malignant behavior. The cell migration assay demonstrated that ART significantly and concentration-dependently inhibited the migration ability of C918 and MUM2B cells (Figure 4D).

These results validated that ART inhibited the proliferation and migration of CM cells in a concentration-dependent manner, preliminary demonstrating the effective inhibitory effect of ART on CM cells. In the subsequent part, we conducted in-depth mechanistic studies based on the key pathways predicted by network pharmacology analysis and the critical genes involved in molecular docking analysis.

### 3.4 ART inhibited the PI3K/AKT/mTOR signaling pathway and promoted cell cycle arrest in CM

**FIGURE 5:**
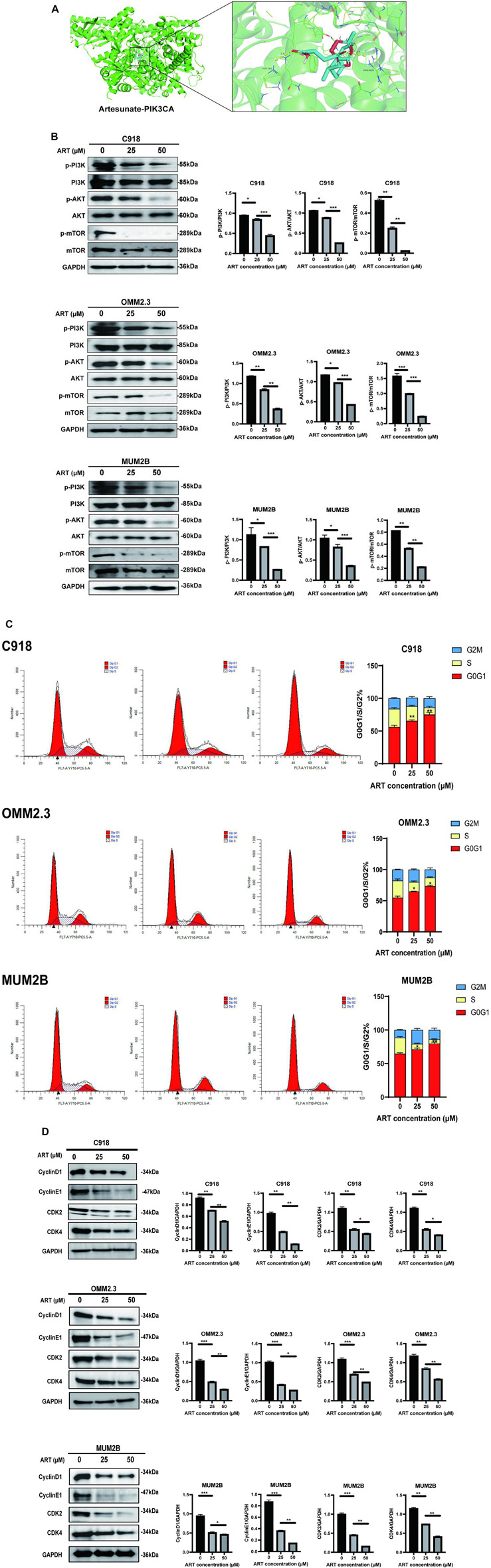
ART exerted its effect of promoting apoptosis by inhibiting the PI3K/AKT/mTOR signaling pathway in CM. **(A)** Molecular docking pattern of ART with PIK3CA. **(B)** WB assays were assessed the expression of p-PI3K, PI3K, p-AKT, AKT, p-mTOR, and mTOR. **(C)**Using flow cytometry to detect the impact of ART on apoptosis with Annexin V/PI dual staining. **(D)** WB assays were assessed the expression of Cyclin D1, Cyclin E1, CDK2, and CDK4.

In this study, molecular docking results indicated that ART can directly interact with multiple amino acid residues (ARG-818 and HIS-759) in the binding pocket of PIK3CA (PI3K) (Figure 5A), demonstrating a strong binding affinity (Table 1). The PI3K/AKT/mTOR signaling pathway is one of the crucial pathways regulating the proliferation process and is considered a key pathway in tumorigenesis (Peng et al., 2022). Based on this inference, it is speculated that ART might exert its anti-proliferation effects by binding to key molecules in this pathway, thereby inhibiting it. Subsequent WB assays confirmed that ART dose-dependently inhibited the expression of this signaling pathway (Figure 5B).

One of the core drivers of cancer cell proliferation is the orderly progression of the cell cycle. Based on this, it is considered whether the inhibitory effects of ART on CM cell proliferation also involve biological processes like cell cycle arrest. After 24 h of ART treatment, flow cytometry assays revealed a significant increase in the proportion of CM cells in the G0/G1 phase and a decrease in the percentage of cells in the S phase, while the proportion of cells in the G2 phase remained unchanged. These results indicated that ART induced G0/G1 phase arrest in CM cells (Figure 5C). It is known that Cyclin D1, which primarily functions in the G1 phase, is a key protein in cell cycle regulation (Chang et al., 2022). It is present in the early G1 phase and forms complexes with CDK4 or CDK6, promoting the transition from the G1 phase to the S phase. Cyclin E binds to and activates CDK2, and the Cyclin E-CDK2 complex is crucial for the progression from the G1 phase to the S phase. WB assays showed that ART induced a dose-dependent decrease in the expression levels of Cyclin D1, Cyclin E1, CDK2 and CDK4 in CM cells (Figure 5D).

Based on the above results, it was speculated that ART inhibited CM cell proliferation by suppressing the PI3K/AKT/mTOR signaling pathway and promoting cell cycle arrest.

### 3.5 ART induced CM apoptosis through the p53 signaling pathway

**FIGURE 6:**
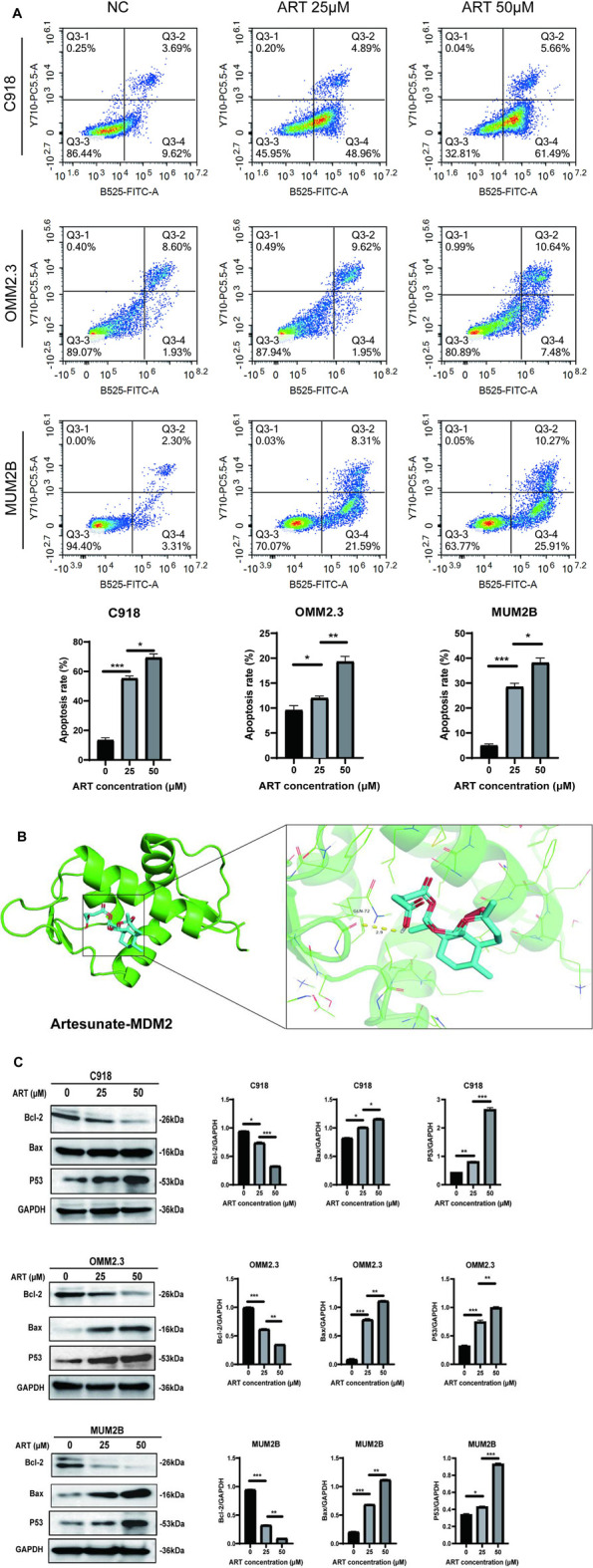
ART exerted its effect of promoting cell cycle arrest by activating the p53 signaling pathway in CM. **(A)** Using flow cytometry to detect impact of ART on cell cycle progression. **(B)** Molecular docking pattern of ART with MDM2. **(C)** WB assays were assessed the expression of Bcl-2, Bax, and p53.

GO and KEGG analyses revealed that ART played a crucial role in the apoptosis pathway of CM cells (Figure 2). Flow cytometry results demonstrated a significant increase in the apoptosis level of CM cells with the increasing concentration of ART treatment (Figure 6A). As a crucial tumor suppressor protein, p53 can upregulate the expression of the pro-apoptotic gene Bax and directly bind to anti-apoptotic proteins like Bcl-2, inhibiting their function, thereby promoting cell apoptosis (Engeland, 2022).

Molecular docking results indicated that ART interacted with the amino acid residue GLN-72 within the binding pocket of MDM2, displaying low binding energy and optimal binding effects (Figure 6B; Table 1). MDM2, as an essential protein, is known to directly bind to p53 and promote its ubiquitination degradation, thereby maintaining the dynamic balance of p53 (Han et al., 2022). Therefore, it can be speculated whether ART induced CM apoptosis through the p53-related pathway. Subsequent WB assays revealed that ART treatment led to an increase in the expression levels of p53 and Bax in CM cells, while the level of Bcl-2 decreased (Figure 6C). Based on the evidence above, it can be strongly speculated that ART induced cell apoptosis by activating the p53 pathway, thereby enhancing the expression of the pro-apoptotic gene Bax and reducing the expression of the anti-apoptotic gene Bcl-2.

### 3.6 ART inhibited CM by promoting oxidative stress and activating the NRF2/HO-1 signaling pathway

**FIGURE 7:**
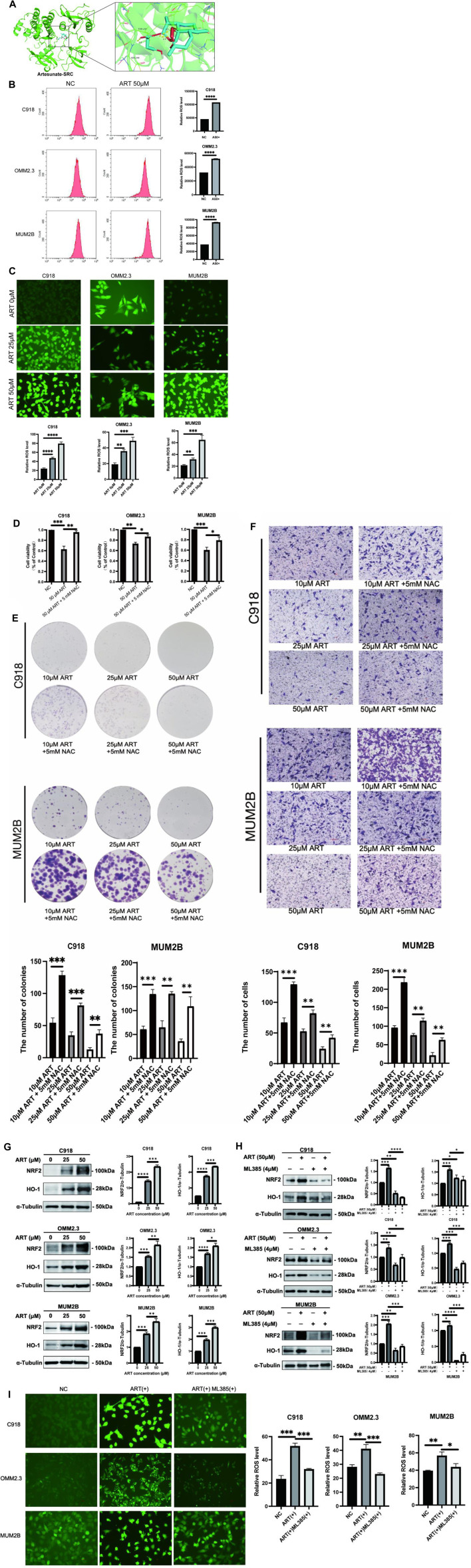
ART enhanced intracellular ROS production and activated the NRF2/HO-1 signaling pathway in CM. **(A)** Molecular docking pattern of ART with SRC. **(B)** Intracellular ROS levels were measured using flow cytometry. **(C)** Intracellular DCFH-DA fluorescence probe content was measured using fluorescence microscopy (scale bar = 50 μm). **(D)** Using CCK-8 assay to test the viability of CM cells which assessed with different concentrations of ART alone or in combination with NAC. **(E)** Using colony formation assay to test the clonogenic ability of CM cells which assessed with different concentrations of ART alone or in combination with NAC. **(F)** Using cell migration assay to test the migration ability of CM cells which assessed with different concentrations of ART and 5 mM NAC (scale bar = 100 μm). **(G)** WB assays were assessed the expression of NRF2 and HO-1. **(H)** WB assays were assessed the differences in the NRF2 and HO-1 expression following treatment with ART alone or in combination with ML385. **(I)** Fluorescence microscopy was used to detect differences in the content of the DCFH-DA fluorescent probe within the cells following treatment with ART alone or in combination with ML385 (scale bar = 50 μm).

In this research, molecular docking results showed that ART can interact well with multiple amino acid residues in the binding pocket of SRC (Figure 7A). In some tumors, activation of SRC has been found to coincide with changes in the level of ROS (Giannoni et al., 2010). ROS level is crucial for regulating cellular processes like apoptosis, cell cycle progression and autophagy in cancer (Cheung and Vousden, 2022). Therefore, it was speculated whether ART exerted its effects against CM through ROS-related pathways.

To further explore the specific mechanism by which ART acts on CM through ROS-related pathways to exert its anti-cancer effects, flow cytometry and fluorescence microscope were used to determine intracellular ROS levels after treatment with ART at different concentrations for 24 h. These findings revealed a significant, concentration-dependent increase in intracellular ROS levels upon ART treatment (Figures 7B,C). Additionally, NAC, as an effective ROS scavenger, partially reversed the proliferation and colony formation of CM induced by ART (Figures 7D,E), as well as its inhibitory effect on cell migration (Figure 7F). These results further confirmed that ART exerted its anti-cancer effects on CM by increasing intracellular ROS levels.

The NRF2/HO-1 signaling pathway is considered a crucial pathway related to oxidative stress. NRF2 is recognized for its ability to relocate to the nucleus and initiate the activation of its target genes, among which HO-1 is included (Lu et al., 2022). This research indicated that after treatment with ART, the protein expression of NRF2 and its downstream gene HO-1 in CM cells was significantly upregulated, with the degree of upregulation increasing with higher ART concentrations (Figure 7G). Rescue experiments were conducted using the NRF2 inhibitor ML385 to further confirm the involvement of the NRF2/HO-1 signaling pathway in the ART-induced oxidative stress process. WB assays demonstrated that the combination of ART and ML385 resulted in a reduction in the expression of NRF2 and its downstream target HO-1 compared to the group treated with ART alone (Figure 7H). Furthermore, experiments using the DCFH-DA probe to detect intracellular ROS showed that when ART was used in combination with ML385, ROS production within the cells showed a notable reduction when compared to the group treated solely with ART, indicating a partial reversal of the intracellular oxidative stress process (Figure 7I). In summary, the above results confirmed that ART exerted its anti-cancer effects by increasing intracellular ROS levels and activating the NRF2/HO-1 signaling pathway.

### 3.7 ART exerted an inhibitory effect on the proliferation of CM *in vivo*

**FIGURE 8:**
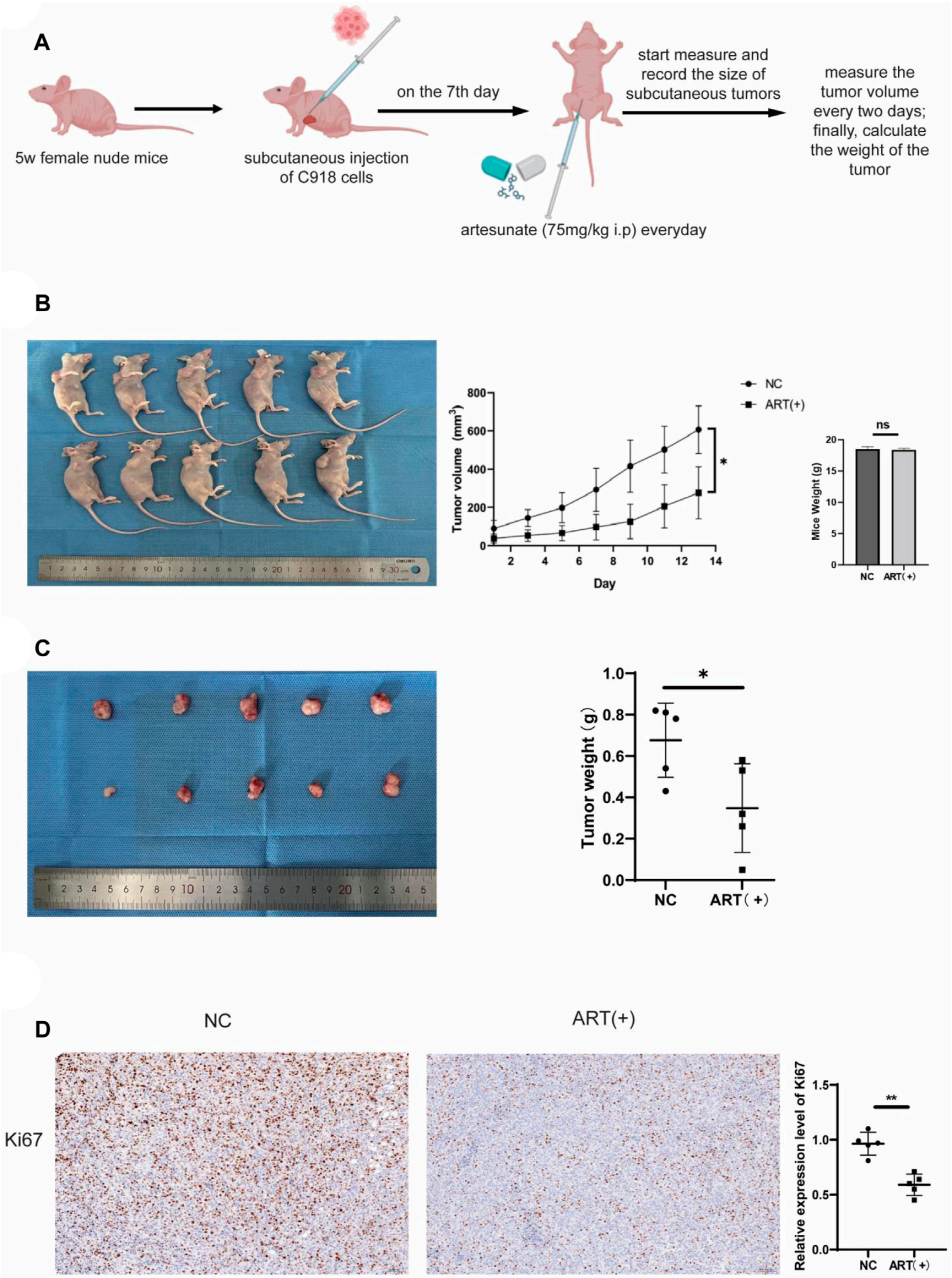
ART inhibited the growth of subcutaneous tumors in nude mice. **(A)** Female nude mice, aged 5 weeks, received subcutaneous injections of 1.5*10^6^ C918 cells in the right axillary region. On the seventh day after tumor injection, the experimental group received daily intraperitoneal injections of 75 mg/kg ART, while the control group received equivalent volumes of physiological saline intraperitoneally. Throughout the 14-days ART treatment period, the body weight and subcutaneous tumor volume of the mice were assessed every 2 days. **(B)** Tumor size was assessed every 2 days during the ART treatment period, and at the conclusion of the 14th days of ART treatment, euthanized the mice, measured the subcutaneous tumors and body weight of the nude mice. **(C)** At the conclusion of the 14th days, measured the size of the tumors and weighed. **(D)** IHC assays were conducted to detect expression of Ki67 in the subcutaneous tumor tissues of the nude mice.

Up to now, through a series of *in vitro* assays, it was confirmed that ART had a significant inhibitory effect on CM. Next, we investigated whether ART treatment could also be effective *in vivo*. As shown in the figure, it was established a timeline to establish a C918 cell-derived tumor model in nude mice and administered the ART (Figure 8A). Observation of the nude mice showed that both the size and weight of tumors in the ART-treated group were lower than those in the control group, while there were no statistically significant differences in the body weight of mice in each group, this confirmed that ART treatment exhibited a significant delay in tumor growth and had no apparent toxic effects on the nude mice (Figures 8B,C). As an important indicator for assessing proliferation, IHC assays showed that the expression of Ki67 in the ART-treated group was significantly higher than that in the control group (Figure 8D). In summary, the above results further confirmed the inhibitory effect of ART on CM *in vivo*.

## 4 Discussion

Currently, due to their diverse biological activities and generally lower side effects compared to synthetic drugs, natural plant extracts are considered an important source of anti-cancer agents (Newman and Cragg, 2020). This research utilized network pharmacology methods to explore the potential anti-cancer mechanisms and core targets of ART against CM, predicted the binding of ART to a series of potential targets through molecular docking techniques and further validated these findings through experiments. The results indicated that ART exerted its anti-cancer effects through multiple pathways, including inhibition of the PI3K/AKT/mTOR pathway, arrest of cell cycle progression, promotion of apoptosis and induction of ROS production.

The PI3K/AKT/mTOR signaling pathway is a potent oncogenic hub that is abnormally activated in various tumors and is closely associated with tumor cell survival and proliferation (Alzahrani, 2019). Molecular docking results in this research confirmed that ART can bind well with PI3K, and WB assays validated that ART inhibited the expression of PI3K/AKT/mTOR signaling pathway, these results provided strong evidence for ART’s inhibitory effect on cell proliferation in CM. In addition, the orderly progression of the cell cycle is crucial for tumor cell proliferation, with the G0/G1 phase being the initial stage. During this phase, cells grow and synthesize necessary RNA and proteins in preparation for DNA replication. This makes the G0/G1 phase a critical stage in tumor development (Engeland, 2022). One of the markers of the G0/G1 phase, Cyclin D1, accumulates in the G1 phase, binds to and activates CDK4/6, driving cells from the G1 phase into the S phase (Chang et al., 2022). Relevant studies confirmed that the PI3K/AKT/mTOR signaling pathway can drive cells from the G1 phase to the S phase by promoting the expression and accumulation of Cyclin D1 (Gao et al., 2004). In summary, we speculated that ART induced G0/G1 phase arrest in CM by inhibiting the PI3K/AKT/mTOR signaling pathway.

In addition to the aforementioned mechanisms, tumors often evade apoptosis through various mechanisms, thereby gaining a survival advantage. Many anti-cancer therapies, such as chemotherapy and radiotherapy, exert their effects by inducing apoptosis in cancer. By promoting apoptosis, these therapies not only inhibit tumor metastasis and proliferation, and eliminate tumor cells, but also overcome drug resistance in patients, thereby improving their prognosis (Su et al., 2015). The regulation of apoptosis involves both intrinsic pathway and extrinsic pathway. Among them, in response to DNA damage and other forms of stress, p53 can upregulate the expression of the pro-apoptotic gene Bax through the intrinsic pathway, promoting cell apoptosis (Ghobrial et al., 2005). Based on this, we speculated that ART promoted apoptosis in CM cells by activating the p53 pathway, accompanied by changes in apoptosis markers. Furthermore, the molecular docking results of this study showed that ART also exhibited good binding interactions with caspase-3 and caspase-8. Caspases are a family of cysteine proteases that play a synergistic role in the cascade of apoptotic signaling. This cascade results in the cleavage of numerous intracellular proteins, followed by cell disassembly, death, and debris clearance. Specifically, caspase-8 can activate downstream caspase effector molecules (such as caspase-3 and caspase-7) in a cascade manner, leading to cell apoptosis (Wieder et al., 2001). This suggested that ART might exert its pro-apoptotic effects in CM through caspase-related pathways, which represented a promising direction for future research.

Additionally, ROS levels also have a significant impact on tumor progression. Under physiological conditions, the intracellular ROS levels are essential for maintaining cellular homeostasis. However, when exceeding a certain threshold, ROS accumulation can induce cellular toxicity, cause DNA damage and ultimately lead to cancer cell death (Schieber and Chandel, 2014; Wang et al., 2020). Artemisinin has been widely used in the treatment of malaria and has been proven to have a high level of drug safety. Its mechanism of action against malaria primarily involves increasing intracellular ROS levels in malaria parasites, leading to cell disintegration and death, thereby exerting its killing effect on target cells (Winzeler and Manary, 2014). As previously reported in other studies, ART can exert its anti-cancer effects by increasing intracellular ROS levels in tumor cells (Zhang et al., 2021). This research also confirmed this anti-cancer mechanism in CM and activated the crucial oxidative stress-related NRF2/HO-1 pathway. When intracellular ROS levels rise, Kelch-like ECH Associated Protein 1 (Keap1) undergoes a conformational change, leading to the release of NRF2 from the Keap1 complex. The released NRF2 then translocates into the nucleus and activates the expression of anti-oxidant genes like HO-1. However, if ROS levels remain excessively high and prolonged, highly activated Keap1 can mediate H_2_O_2_-induced oxeiptosis through an NRF2-independent mechanism, resulting in cell death (Tang et al., 2019). Oxeiptosis is a type of cell death characterized by a ROS-induced, caspase-independent and apoptosis-like pathway (Holze et al., 2018). This process is distinct from other forms of programmed cell death, such as apoptosis and autophagy, and it specifically relies on ROS-mediated signaling. Based on these findings, ART exerted its anti-cancer effect on CM through oxeiptosis and activated the NRF2/HO-1 signaling pathway. Given its pharmacological mechanism similar to that in anti-malarial treatment, it is speculated that utilizing ART to increase intracellular oxidative stress levels to inhibit CM is highly feasible and safe.

Cell migration assays have demonstrated that ART significantly inhibited the migration capacity of CM cells, and molecular docking results also revealed that ART can bind well with MMP9 and MMP2. Matrix Metalloproteinases (MMPs) are a class of zinc-dependent endopeptidases capable of degrading various components of the extracellular matrix (ECM) (Olivares-Urbano et al., 2020), facilitates tumor cells in breaching the basement membrane and matrix barriers, thereby migrating to other sites, and is considered a key factor in tumor metastasis, such as breast cancer (Olivares-Urbano et al., 2020), lung cancer (Merchant et al., 2017) and gastric cancer (Sokolova and Naumann, 2022). Based on this, we speculated that ART might also inhibit CM cell migration by targeting MMPs, which represented a promising direction for our future research.

In summary, we have elaborated on how ART exerted its inhibitory effects by targeting oncogenic pathways, including promoting apoptosis, inducing cell cycle arrest and increasing oxidative stress levels in CM. Further research is needed to explore whether ART induces apoptosis in CM through the Caspase pathway, as well as its role in promoting CM migration through MMPs.

## 5 Conclusion

This study employed a comprehensive approach combining network pharmacology, molecular docking and experimental validation, confirming that the significant anti-cancer effect of ART on CM may be attributed to its effective inhibition of multiple key mechanisms in tumorigenesis. These findings not only revealed the significant potential of natural compounds in clinical oncology treatment but also offer hope for significant advancements in early prevention and control of CM.

## Data Availability

The original contributions presented in the study are included in the article/Supplementary Material, further inquiries can be directed to the corresponding authors.
